# Supplementary material for: The effect of a behaviour change intervention on the diets and physical activity levels of women attending Sure Start Children’s Centres: results from a complex public health intervention
Source: BMJ Open. 2014 Jul 15;4(7):e005290. doi: 10.1136/bmjopen-2014-005290 (PMC4120404; doi:10.1136/bmjopen-2014-005290)
Supplement: Author's manuscript [file bmjopen-2014-005290.draft_revisions.pdf]

PEER REVIEW HISTORY

BMJ Open publishes all reviews undertaken for accepted manuscripts. Reviewers are asked to complete a checklist review form (**http://bmjopen.bmj.com/site/about/resources/checklist.pdf**) and are provided with free text boxes to elaborate on their assessment. These free text comments are reproduced below. Some articles will have been accepted based in part or entirely on reviews undertaken for other BMJ Group journals. These will be reproduced where possible.

ARTICLE DETAILS

**TITLE (PROVISIONAL)**	The effect of a behaviour change intervention on the diets and physical activity levels of women attending Sure Start Children's Centres: results from a complex public health intervention
**AUTHORS**	Baird, Janis; Jarman, Megan; Lawrence, Wendy; Black, Christina; Davies, Jenny; Tinati, Tannaze; Begum, Rufia; Mortimore, Andrew; Robinson, S; Margetts, Barrie; Cooper, Cyrus; Barker, Mary; Inskip, Hazel

VERSION 1 - REVIEW

**REVIEWER**	Dr Sanjoti Parekh<br>Griffith University, Queensland, Australia
**REVIEW RETURNED**	27-May-2014

**GENERAL COMMENTS**	1. Figure 1 , 2 ,3 and 4 are unnecessary.<br>2. Page 15, last sentence seems to be incomplete<br>3. Presentation of results related to improvement in physical activity seem incorrect as there was no change in comparison with change in control group. 4. It is not clear how higher education level or workforce involvement has impacted the final results.

**REVIEWER**	Dr Stephen-Mark Cooper<br>Cardiff School of Sport<br>Cardiff Metropolitan University<br>Cyncoed Campus<br>Cyncoed Road<br>Cyncoed
**REVIEW RETURNED**	31-May-2014

**GENERAL COMMENTS**	I really enjoyed reading your paper. It was contemporary and timely and you kept my interest throughout. A good example of practical and applied research. You might want to give some thought to differentiating between statistically significant outcomes/effects and practically significant outcomes. Thank you for the opportunity to review it.<br><br>In this manuscript (MS) the authors apply a set of appropriate statistical tests by way of evaluating the effects of an intervention strategy aimed at improving dietary quality and levels of physical activity in a total of n = 527 disadvantaged women attending Sure Start Children's Centres (SSCC) in Southampton and a control group of n = 495 women in Gosport and Havant. The study was

	designed as a non-random control trial.<br><br>In my view the study is well-designed, the research is well-organised and the data have been collected appropriately. On the whole the level of detail provided is very good. As far as the methodology is concerned I am convinced that the study could be replicated based on the description the authors provide in the MS. Generally, the authors communicate their arguments in a clear and concise manner in a paper that I found interesting, relevant and contemporary. It is well-crafted and skilfully written.<br><br>Sometimes there is inconsistency in the expression of terms and the MS could also do with proof reading in terms of item spacing. My specific comments are as follows where I have used the page numbers and line numbers appended by the BMJ Open review software:<br><br>**Title**: In my view the authors should give some thought to re-writing the title. It is clumsy and overlong. It should be shorter and punchier.<br><br>**Abstract (page 3)**<br><br>**Line 8:** … UK governmen**t's** response …<br><br>**Line 10:** … This study evaluated **the** effects of …<br><br>**Line 11:** and elsewhere throughout the MS starting with the title … a better term might be **levels of physical activity** … so, … dietary quality and **levels of physical activity in** women from …<br><br>**Line 24:** are the 'Centres' referred to in Gosport and Havant SSCCs? If so say so.<br><br>**Line 36:** as above … quality and **levels of physical activity**. I won't mention this again. Also, and throughout the MS, self-efficacy is written correctly in this sentence (with the hyphen) but that is not always the case in the rest of the MS.<br><br>**Lines 40-58 (results section):** the authors mention the terms 'significant' and 'significantly' in this section of the abstract. I'm assuming this means statistical significance rather than practical significance or clinical significance. If so, they should distinguish this. What were the significance levels (*P*-values)? They have 95%CIs but what were the sample point estimates (means or medians)? It might be better to express the confidence intervals as **95%CI: 0.001 to 0.50** for example. On line 48 it should be 'self-efficacy' with the hyphen.<br><br>**Page 4, line 5:** … and levels of physical activity, it did have a protective …<br><br>**Line 9:** and throughout the MS … intervention **might** improve … 'may' is a request.<br><br>**Introduction (page 6)**

Line 9: I suggest italicising '*Healthy Lives, Healthy People*'

Line 12: … population improve**s**. …

Line 18: … influence over food-related decisions …

Line 22: … in postnatal life. Growth and development …

Line 27: … likely to have poor quality diets and …

Line 36: I don't think that the sentence makes sense with the word 'prospective' in it. Prospective is an adjective that means in the future. It should be omitted.

Line 37: and extensively elsewhere throughout the MS the authors need to be consistent in spacing their citation numbers and the start of new sentences. Also, … of mother**s'** diets and …

Line 44: … Data from **the** SWS have …

Line 49: … Studies **exploring** barriers to …

Line 51: … diets felt **that** they lacked …

Line 53: and elsewhere in the MS – the authors refer to 'associations' when the likelihood is that the statistics used in the analysis was a correlation – in which case the correct term is 'relationship' or 'relationships'. The Americans use association when they mean relationship. So, … confirmed the **relationship between a** higher sense of control and self-efficacy with …

Page 7, lines 3-8: this is one sentence! The authors are very fond of overlong sentences in this MS!

Line 14: … self-monitoring. **This** review … frequent contact with the …

Line 19: …from population-based studies …

Line 23: … disease self-management …

Line 26: it might be useful for the authors to explain briefly how they are using the term 'self-efficacy' in this MS – a definition in this context.

<u>Methods</u> (page 8)

Line 11: I don't think there is a need for the quote marks … The healthy conversation skills (HCS) training …

Page 9, line 3: and elsewhere throughout the MS – the authors use 'prior to' when they mean 'before'. So … Gosport and Havant **before** the start …

Line 13: the Southampton Women's Survey has been initialised previously to SWS so use the initialisation or don't initialise any terms and write them in full at every mention.

Line 15: … produce standardised scores (*z*-scores with mean 0.0

	and standard deviation 1.0).[25] …<br><br>**Line 18:** … not feasible. **Level of physical activity** was …<br><br>**Line 20:** … (GPPAQ) which allows **for** categorisation of …<br><br>**Line 30:** … was derived from mother**s'** response**s** to …<br><br>**Lines 12-35:** measurement reliability and validity is population specific. To what degree are the authors confident that the data collection methods described in the present MS are valid and reliable for use with the population of interest here? I suspect that most of these inventories were developed in north America – how valid are they with disadvantaged women living in Southampton, Gosport and Havant?<br><br>**Line 43:** and elsewhere throughout the MS … baseline and **follow-up** …<br><br>**Line 49:** had given **previously** at baseline. …<br><br>**Line 54:** what were the other 'covariates' referred to?<br><br>**Page 10, lines 22-34 (statistical analysis section):** I don't have a major issue with the statistical tests used to analyse the data but I think more needs to be made here to clarify why certain tests were deemed appropriate. Where independent *t*-tests (note use of italics) were used, did the authors make the judgement in view of confirmed normality of saved residuals and homogeneity of variance about the means? What was the level of statistical significance set for the data analysis and why? What type of error were the authors trying to avoid? Later in the MS, in the tables, Wilcoxon's test is mentioned as are odds ratios but no reference is made to either statistic here. Additionally there is no mention of quantifying 'practical significance' – had the authors considered including some measure of effect size in the MS?<br><br>**<u>Results</u> (page 10)**<br><br>**Line 44:** and elsewhere in the MS … shown in **T**able 1. …<br><br>**Table 1, page 11:** and elsewhere in the MS … *n* = 243 … *n* = 266 … *P*-value … Age is measured in 'yrs' … in the body of the table italicise all *n* … note italics and spacing<br><br>**Table 1, page 12:** reference to (RR) on PAI – is this relative risk? If so there's no mention of it in the methods section. What is SD in the prudent diet score?<br><br>**Page 12, lines 25-36:** there are percentages presented as whole numbers and as fractions – in which case … 36.0%, 85.0% and 63.0%.<br><br>**Line 48:** … intervention group (*P* = 0.01 for … note italics and spacing<br><br>**Table 2, page 13:** reference to (RR) on PAI – is this relative risk? What is SD in the prudent diet score? … *P*-value … In the key below

	the table – it is Wilcoxon and *t*-test<br><br>**Line 51:** it is interesting to note that the 95%CI is expressed here as I suggested above!<br><br>**Page 14, line 34:** … 'related to' not 'associated with' … the test is a correlation/regression.<br><br>**Line 36:** and elsewhere throughout the MS … (β = 0.1126, 95%CI: 0.718 to 0.1535, P < 0.001 and … note consistent use of italics, spacing and capital letters. Also, you can't have P = 0.000 – this is actually P = 0.00005.<br><br>**Line 38:** if the test was correlation it is 'relationship' not 'association'<br><br>**Line 43:** past tense … which **was** highly correlated …<br><br>**Line 44:** … decline (***β*** = 0.0784, **why no 95%CI**, ***P*** = 0.001), the **relationship** being …<br><br>**Page 15, line 5:** … statistically significant ***P* = ? (Table 2).** …<br><br>**Line 8:** …form of plot as **that** in Figure 1 …<br><br>**Line 13:** and elsewhere in the MS … There were **non**-significant differences …<br><br>**Page 16, line 36:** … the relationship**s** of … 18 months **before** follow-up) …<br><br>**Line 41:** a common error – there must have been some relationship between the variables – what the authors mean is … there was **a non-significant relationship** between …<br><br>**Page 17, line 43:** … significant (***P* > 0.05**. Table 2 … note use of italics and spacing<br><br>**Line 49:** … **follow-up that might** have … not 'may'<br><br>**Line 52:** … baseline and **follow-up** (**T**able 3). …<br><br>**Page 18, line 5:** … as shown in **T**able 3 …<br><br>**Line 8:** … 23.5% (***P* = 0.02**). …<br><br>**Table 3:** … (*n*(%)) … *P*-value … In the key below the table – it is Wilcoxon<br><br>**Line 44:** … 'related to' not 'associated with' … the test is a correlation/regression<br><br>**Line 49:** … **follow-up** (***β*** = 0.7683, **95%CI: 0.5893 to 0.9673**, ***P* < 0.001**) …<br><br><br>**Discussion (page 19)**

	**Line 14:** … There were **non-**significant differences …<br><br>**Line 38:** suggest … demonstrated that **improved consumption of fruit and vegetables** are mediated …<br><br>**Line 44:** and elsewhere in the MS – there is no point in initialising WIC if you are not going to use it again in the MS.<br><br>**Line 51:** … similar approach to the **present study's** intervention …<br><br>**Page 20, line 5:** … a trained **clinician** was …<br><br>**Line 11:** … **The present study** was innovative …<br><br>**Line 24:** … that they **can** continue … not 'may'<br><br>**Lines 32-36:** this is a rather clumsy sentence. What does the reference to 'natural experiments' mean?<br><br>**Line 38:** … The design of **the present** complex …<br><br>**Line 40 & 48 & 51:** and elsewhere in the MS – it has been SSCC previously – either continue to use the initialisation or dispense with it and use the full name in every instance and with every name<br><br>**Line 52:** … Southampton, and in Gosport and Havant …<br><br>**Page 21, line 3:** … attainment than **the** women in the …<br><br>**Line 6:** … owned their **own** home. …<br><br>**Line 12:** … **Another strength of** … I also go back to my point earlier about validity being population specific. These measures might have been validated for a given population but was that the same population as those disadvantaged women used in the present study from Southampton, Gosport and Havant?<br><br>**Lines 12-17:** another overlong and clumsy sentence – it needs reworking<br><br>**Line 25:** … decline in the **18** month …<br><br>**Line 27:** … and **follow-up** survey …<br><br>**Line 31:** … intervention group **than in** the control …<br><br>**Line 39 & 45: might** not may<br><br>**Line 40:** … among the **study's participants** …<br><br>**Line 53:** … there **were non-significant relationships** between …<br><br>**Page 22, lines 8, 20 & 22: might** not may<br><br>**Line 31:** again … it has been SSCC previously – either continue to use the initialisation or dispense with it and use the full name in every instance and with every name – keep it consistent

	**Page 23, lines 3, 5, 35 & 54: might** not may<br><br>**Line 20:** … for the relationship**s** between …<br><br>**Line 45:** … of carrying **out** complex …<br><br>**Page 24, line 3 (twice): might** not may<br><br>**Line 17:** consistent in spacing citation numbers and the start of new sentences<br><br>**Line 24:** … community **level** intervention …<br><br><br>**References (page 26)**<br><br>There is a lack of consistency here. Some journals are named in full whilst others are abbreviated. I'm not sure what BMJ Open's policy on this is but I suggest all journal names are written in full.<br><br>Reference 24 – has this citation now been made available in print 2014?<br><br>Reference 40 – it is the **International** Journal of Pediatric Obesity<br><br>Reference 42 – I could not find a citation number 42 in the text – perhaps I missed it

VERSION 1 – AUTHOR RESPONSE

Reviewer 1

1. Figure 1, 2 ,3 and 4 are unnecessary.
Our response: We have deleted the figures and any accompanying text but have ensured that points about the size or direction of a relationship are still made in the text with reference to the relevant table.

2. Page 15, last sentence seems to be incomplete
Our response: This partial sentence should have been deleted before submission. It has been deleted now.

3. Presentation of results related to improvement in physical activity seem incorrect as there was no change in comparison with change in control group.
Our response: We have described statistically significant improvements within the intervention and control groups but have made it clear that there was a non-significant difference in the change between the groups. This relevant text is in the results section of the abstract and on page 18 of the track-changed version of the paper. We wonder whether the reviewer might have overlooked some of this text.

4. It is not clear how higher education level or workforce involvement has impacted the final results.

Our response: We describe the influence of level of educational attainment on the decline in dietary

quality between baseline and follow-up – it was the strongest predictor of the decline, independent of the effects of food security, receipt of benefits, sense of control and self-efficacy. The smaller reductions in self-efficacy and sense of control in the intervention compared with the control group were statistically significant and were independent of the effect of educational attainment (table 2) - this analysis used regression models adjusted for baseline levels of variables thus taking account of confounding factors that relate equally to baseline and follow-up measures, including educational attainment. We were not powered to look at interactions between the intervention and educational attainment. These findings are described in the results section and discussed within the interpretation of findings in the discussion section.

There was a statistically significant increase in the proportion of women in paid work between baseline and follow-up. The decline in dietary quality was not explained by this but being in work was strongly related to increased levels of physical activity. These findings are presented within the results section and discussed within the interpretation of findings in the discussion section.

Reviewer 2

1. I really enjoyed reading your paper. It was contemporary and timely and you kept my interest throughout. A good example of practical and applied research. You might want to give some thought to differentiating between statistically significant outcomes/effects and practically significant outcomes. Thank you for the opportunity to review it.

Our response: We are grateful to reviewer 2 for his positive comments on our study and we appreciate the meticulous approach he has taken to suggesting corrections throughout the manuscript. We are grateful for the reviewer's suggestion that we should differentiate between statistical and practical significance. We have added text to the discussion section to explain better the practical significance of the sizes of effect that we observed in relation to self-efficacy and sense of control – page 20 of the track-changed version of the manuscript.

2. In this manuscript (MS) the authors apply a set of appropriate statistical tests by way of evaluating the effects of an intervention strategy aimed at improving dietary quality and levels of physical activity in a total of n = 527 disadvantaged women attending Sure Start Children's Centres (SSCC) in Southampton and a control group of n = 495 women in Gosport and Havant. The study was designed as a non-random control trial.

In my view the study is well-designed, the research is well-organised and the data have been collected appropriately. On the whole the level of detail provided is very good. As far as the methodology is concerned I am convinced that the study could be replicated based on the description the authors provide in the MS. Generally, the authors communicate their arguments in a clear and concise manner in a paper that I found interesting, relevant and contemporary. It is well-crafted and skilfully written. Sometimes there is inconsistency in the expression of terms and the MS could also do with proof reading in terms of item spacing. My specific comments are as follows where I have used the page numbers and line numbers appended by the BMJ Open review software:

Title: In my view the authors should give some thought to re-writing the title. It is clumsy and overlong. It should be shorter and punchier.

Our response: We have amended the title which is now: What is the effect of a behaviour change intervention on the diets and levels of physical activity of women attending Sure Start Children's Centres?

3. Abstract (page 3)
Line 8: 3 UK government's response
Our response: This change has been made.

4. Line 10: 3 This study evaluated the effects of
Our response: This change has been made.

5. Line 11: and elsewhere throughout the MS starting with the title 3 a better term might be levels of physical activity so, dietary quality and levels of physical activity in women from

Our response: This change has been made throughout the manuscript, including the title.

6. Line 24: are the 'Centres' referred to in Gosport and Havant SSCCs? If so say so.
Our response: This change has been made.

7. Line 36: as above quality and levels of physical activity. I won't mention this again. Also, and throughout the MS, self-efficacy is written correctly in this sentence (with the hyphen) but that is not always the case in the rest of the MS.

Our response: As above in point 5 – this change has been made throughout the manuscript.

8. Lines 40-58 (results section): the authors mention the terms 'significant' and 'significantly' in this section of the abstract. I'm assuming this means statistical significance rather than practical significance or clinical significance. If so, they should distinguish this. What were the significance levels (P-values)? They have 95%CIs but what were the sample point estimates (means or medians)? It might be better to express the confidence intervals as 95%CI: 0.001 to 0.50 for example. On line 48 it should be 'self-efficacy' with the hyphen.

Our response: We have amended the text throughout to make it clear when we are referring to statistical significance and have reported P-values. We have amended our text to be consistent with BMJ Open's approach to CIs, which is '95% CIs x to y'.

9. Page 4, line 5: 3 and levels of physical activity, it did have a protective
Line 9: and throughout the MS intervention might improve 'may' is a request.

Our response: This change has been made throughout the manuscript.

10. Introduction (page 6)
Line 9: I suggest italicising 'Healthy Lives, Healthy People'
Line 12: population improves.
Line 18: influence over food-related decisions
Line 22: in postnatal life. Growth and development
Line 27: likely to have poor quality diets and
Line 36: I don't think that the sentence makes sense with the word 'prospective' in it. Prospective is an adjective that means in the future. It should be omitted.
Line 37: and extensively elsewhere throughout the MS the authors need to be consistent in spacing their citation numbers and the start of new sentences. Also, of mothers' diets and
Line 44: Data from the SWS have

Line 49: Studies exploring barriers to
Line 51: diets felt that they lacked

Our response: The above corrections to the introduction have all been made.

11. Line 53: and elsewhere in the MS – the authors refer to 'associations' when the likelihood is that the statistics used in the analysis was a correlation – in which case the correct term is 'relationship' or 'relationships'. The Americans use association when they mean relationship. So, confirmed the relationship between a higher sense of control and self-efficacy with

Our response: We appreciate the point that the reviewer is making and have amended the text throughout the manuscript replacing 'association' with 'relationship' where appropriate.

12. Page 7, lines 3-8: this is one sentence! The authors are very fond of overlong sentences in this MS!
Our response: This sentence has now been divided into two shorter sentences.

13. Line 14: self-monitoring. This review frequent contact with the
Line 19: from population-based studies
Line 23: disease self-management

Our response: These corrections have been made.

14. Line 26: it might be useful for the authors to explain briefly how they are using the term 'self-efficacy' in this MS – a definition in this context.

Our response: We have added definitions of sense of control and self-efficacy to the description of the conceptual model within the introduction.

15. Methods (page 8)
Line 11: I don't think there is a need for the quote marks ... The healthy conversation skills (HCS) training
Our response: We have removed the quotation marks and instead used capital letters for Healthy Conversation Skills (HCS).

16. Page 9, line 3: and elsewhere throughout the MS – the authors use 'prior to' when they mean 'before'. So Gosport and Havant before the start

Our response: We have replaced 'prior to' with 'before' throughout the manuscript.

17. Line 13: the Southampton Women's Survey has been initialised previously to SWS so use the initialisation or don't initialise any terms and write them in full at every mention.
Line 15: produce standardised scores (z-scores with mean 0.0 and standard deviation 1.0).25
Line 18: not feasible. Level of physical activity was
Line 20: (GPPAQ) which allows for categorisation of
Line 30: was derived from mothers' responses to

Our response: The above changes relating to lines 13 to 30 of the methods section have been made.

18. Lines 12-35: measurement reliability and validity is population specific. To what degree are the authors confident that the data collection methods described in the present MS are valid and reliable for use with the population of interest here? I suspect that most of these inventories were developed in north America – how valid are they with disadvantaged women living in Southampton, Gosport and Havant?

Our response: The majority of the measures we used were developed for, or have been previously used in UK populations. This includes the 20-item FFQ which was developed in Southampton, UK, the self-efficacy scale, which has been widely used throughout the world including the UK, and the GPPAQ which has been recommended for UK populations by NICE. The scale we used for sense of control was used in the Whitehall II Study in which a third of the participants were women aged 35 to 55. So although this is a UK population, the women taking part were older than those taking part in the present study. Before embarking on the study we assessed the face validity of measures with women in Southampton. This pilot work led to the selection of the GPPAQ in preference to the IPPAQ or Recent PAQ. We have amplified the strengths and weaknesses section of the discussion, on page 22 of the track-changed version of the manuscript, to make issues of validity more transparent.

19. Line 43: and elsewhere throughout the MS baseline and follow-up
Line 49: had given previously at baseline.
Our response: These amendments have been made.

20. Line 54: what were the other 'covariates' referred to?
Our response: The covariates are the intermediate outcomes – self-efficacy and sense of control. We can see that the use of the term 'covariates' does not make this clear. We have amended the text to:.....'levels of main and intermediate outcomes'...

21. Page 10, lines 22-34 (statistical analysis section): I don't have a major issue with the statistical tests used to analyse the data but I think more needs to be made here to clarify why certain tests were deemed appropriate. Where independent t-tests (note use of italics) were used, did the authors make the judgement in view of confirmed normality of saved residuals and homogeneity of variance about the means? What was the level of statistical significance set for the data analysis and why? What type of error were the authors trying to avoid? Later in the MS, in the tables, Wilcoxon's test is mentioned as are odds ratios but no reference is made to either statistic here. Additionally there is no mention of quantifying 'practical significance' – had the authors considered including some measure of effect size in the MS?

Our response: We thank the reviewer for pointing out that our analysis description needed more detail; we had kept it short as the paper is lengthy and some of our additional analysis methods were mentioned in the footnotes of the tables. We agree that a fuller description would be helpful and so have revised the statistical analysis section to describe the details of our analyses in one section and to include the issues raised by the reviewer. We have amended Table 2 slightly, having noted some small rounding errors, and reanalysed the Physical Activity Level data using Poisson regression, which we now believe is more appropriate for these data not least as it provides relative risks rather than odds ratios; the method is described in the text. We have also added a comment about the level of statistical significance we use to describe results as non-significant.

22. Results (page 10)
Line 44: and elsewhere in the MS shown in Table 1.
Table 1, page 11: and elsewhere in the MS n = 243 n = 266 P-value Age is measured in 'yrs' in the body of the table italicise all n note italics and

spacing
Our response: These changes have been made.

23. Table 1, page 12: reference to (RR) on PAI – is this relative risk? If so there's no mention of it in the methods section. What is SD in the prudent diet score?
Our response: We have added a description of the relative risk calculation for PAI to the methods, statistical analysis section on page 10.

24. Page 12, lines 25-36: there are percentages presented as whole numbers and as fractions – in which case 36.0%, 85.0% and 63.0%.
Line 48: 3 intervention group (P = 0.01 for 3 note italics and spacing
Table 2, page 13: reference to (RR) on PAI – is this relative risk? What is SD in the prudent diet score? P-value In the key below the table – it is Wilcoxon and ttest
Line 51: it is interesting to note that the 95%CI is expressed here as I suggested above!
Our response: These changes have been made.

25. Page 14, line 34: 'related to' not 'associated with' the test is a correlation/regression.
Line 36: and elsewhere throughout the MS (β = 0.1126, 95%CI: 0.718 to 0.1535, P < 0.001 and note consistent use of italics, spacing and capital letters. Also, you can't have P = 0.000 – this is actually P = 0.00005.
Line 38: if the test was correlation it is 'relationship' not 'association'
Line 43: past tense which was highly correlated
Line 44: decline (β = 0.0784, why no 95%CI, P = 0.001), the relationship being

Our response: The corrections have been made. In relation to spacing between P and B with equals signs, we have checked articles published in BMJ Open and the journal does not use spaces between P and β and the equals sign. So we have not changed our text.

26. Page 15, line 5: statistically significant P = ? (Table 2).
Line 8: form of plot as that in Figure 1
Line 13: and elsewhere in the MS There were non-significant differences
Page 16, line 36: the relationships of 18 months before follow-up)
Line 41: a common error – there must have been some relationship between the variables – what the authors mean is there was a non-significant relationship between
Our response: These changes have been made.

27. Page 17, line 43: significant (P > 0.05. Table 2 note use of italics and spacing
Line 49: follow-up that might have not 'may'
Line 52: baseline and follow-up (Table 3).
Page 18, line 5: as shown in Table 3
Line 8: 23.5% (P = 0.02).
Table 3: (n(%)) P-value In the key below the table – it is Wilcoxon
Line 44: 'related to' not 'associated with' the test is a correlation/regression
Line 49: follow-up (β = 0.7683, 95%CI: 0.5893 to 0.9673, P < 0.001)
Discussion (page 19)
Line 14: There were non-significant differences
Line 38: suggest demonstrated that improved consumption of fruit and vegetables are mediated

Our response: These changes have been made

28. Line 44: and elsewhere in the MS – there is no point in initialising WIC if you are not going to use it again in the MS.

Our response: Many people only know the WIC intervention by its acronym. We have therefore retained the acronym and would be grateful if BMJ editors would make a judgement on this point.

29. Line 51: similar approach to the present study's intervention
Page 20, line 5: a trained clinician was
Line 11: The present study was innovative
Line 24: that they can continue not 'may'
Our response: These changes have been made

30. Lines 32-36: this is a rather clumsy sentence. What does the reference to 'natural experiments' mean?
Our response: While the present study is not a natural experiment, there are many similarities with one in the sense that we did not have control over the processes within SSCCs that might bring participants into contact with trained SSCC staff. We have amplified this point within the manuscript.

31. Line 38: The design of the present complex
Line 40 & 48 & 51: and elsewhere in the MS – it has been SSCC previously – either continue to use the initialisation or dispense with it and use the full name in every instance and with every name
Line 52: Southampton, and in Gosport and Havant
Page 21, line 3: attainment than the women in the
Line 6: owned their own home.
Line 12: Another strength of I also go back to my point earlier about validity being population specific. These measures might have been validated for a given population but was that the same population as those disadvantaged women used in the present study from Southampton, Gosport and Havant?

Our response: These changes have been made and we have rewritten the sentence on validity making it clear that not all the measures we used had been validated for our study population.

32. Lines 12-17: another overlong and clumsy sentence – it needs reworking

Our response: The sentence had been divided into two shorter sentences.

33. Line 25: decline in the 18 month
Line 27: and follow-up survey
Line 31: intervention group than in the control
Line 39 & 45: might not may
Line 40: among the study's participants
Line 53: there were non-significant relationships between
Page 22, lines 8, 20 & 22: might not may
Line 31: again it has been SSCC previously – either continue to use the initialisation or dispense with it and use the full name in every instance and with every name – keep it consistent
Page 23, lines 3, 5, 35 & 54: might not may
Line 20: for the relationships between

Line 45: of carrying out complex
Page 24, line 3 (twice): might not may

Our response: These changes have been made and SSCC is now used throughout the paper with the exception of its first mention in the paper, when it has been given in full.

34. Line 17: consistent in spacing citation numbers and the start of new sentences
Line 24: community level intervention
Our response: These changes have been made.

35. References (page 26)
There is a lack of consistency here. Some journals are named in full whilst others are abbreviated. I'm not sure what BMJ Open's policy on this is but I suggest all journal names are written in full.
Reference 24 – has this citation now been made available in print 2014?
Reference 40 – it is the International Journal of Pediatric Obesity
Reference 42 – I could not find a citation number 42 in the text – perhaps I missed it
Our response: These corrections have been made. In relation to the references, one of the studies (Spanou et al) should have been removed as it has been superseded by a more recent publication (Butler et al). We have also added a new reference in response to the point about the practical significance of our findings. The total number of references is therefore 42.
